# Tele-Neuropsychological Assessment of Alzheimer’s Disease

**DOI:** 10.3390/jpm11080688

**Published:** 2021-07-21

**Authors:** Anna Carotenuto, Enea Traini, Angiola Maria Fasanaro, Gopi Battineni, Francesco Amenta

**Affiliations:** 1 Centre for Clinical Research, Telemedicine and Telepharmacy, University of Camerino, Via Madonna delle Carceri 9, 62032 Camerino, Italy; annacarotenuto@gmail.com (A.C.); enea.traini@unicam.it (E.T.); angiolamfasanaro@gmail.com (A.M.F.); francesco.amenta@unicam.it (F.A.); 2 Research Department, International Radio Medical Centre (C.I.R.M.), Via dell’Architettura 41, 00144 Roma, Italy

**Keywords:** telemedicine, dementia, AD, neuropsychological tests, videoconferencing

## Abstract

Background: Because of the new pandemic caused by the novel coronavirus disease (COVID-19), the demand for telemedicine and telemonitoring solutions has been exponentially raised. Because of its special advantage to treat patients in an emergency without physical presence at a hospital via video conferencing, telemedicine has been used to overcome distance barriers and to improve access to special domains like neurology. In these pandemic times, telemedicine has been also employed as a support for the diagnosis and treatment of adult-onset dementia disorders including Alzheimer’s disease. Objective: In this study, we carried out a systematic literature analysis to clarify if the neuropsychological tests traditionally employed in face-to-face (FTF) contexts are reliable via telemedicine. Methods: A systematic literature search for the past 20 years (2001–2020) was carried out through the medical databases PubMed (Medline) and the Cumulative Index to Nursing and Allied Health Literature (CINAHL). The quality assessment was conducted by adopting the Newcastle Ottawa Scale (NOS) and only studies with a NOS ≥ 7 were included in this review. Results: The Mini-Mental State Examination (MMSE) results do not differ when tests are administered in the traditional FTF modality or by videoconference, and only negligible minor changes in the scoring system were noticeable. Other neuropsychological tests used to support the diagnosis of AD and dementia such as the Token Test, the Comprehension of Words and Phrases (ACWP), the Controlled Oral Word Association Test showed high reliability between the two modalities considered. No differences in the reliability concerning the living setting or education of the subjects were reported. Conclusions: The MMSE, which is the main screening test for dementia, can be administered via telemedicine with minor adaptation in the scoring system. Telemedicine use for other neuropsychological tests also resulted in general reliability and enough accuracy. Cognitive assessment by videoconference is accepted and appreciated and therefore can be used for dementia diagnosis in case of difficulties to performing FTF assessments. This approach can be useful given a personalized medicine approach for the treatment of adult-onset dementia disorders.

## 1. Introduction

Telemedicine has been used to overcome distance barriers and to improve access to medical services that would often not be consistently available in remote rural communities and areas of difficult access [1]. Telemedicine includes all medical activities in diagnosis, therapeutics, or social medicine undertaken using electronic transfer media, and enabling the transmission of visual and acoustic information over long distances without the need that a doctor is personally present at the requested consultation [1,2].

Telehealth technologies permit communication between a patient and medical staff with both convenience and fidelity, as well as the transmission of medical, imaging, and health data from one site to another [3]. From the first use, dating back to 1920, when telemedicine helped to assist people in case of medical emergencies on board ships, it had a progressive and significant development, and at the present, it encompasses more than 50 different subspecialties [1,4].

Dementia is a chronic disease characterized by a cognitive decline in progressive nature [5]. Most of the adult-onset dementia disorders (60–80%) are associated with Alzheimer’s disease (AD) and followed by cerebrovascular disease (vascular dementia), Lewy body disease, Parkinson’s disease (PD), and others [6]. Patients with AD gradually lose memory, thinking abilities, decision making, and language. The cognitive assessment of older subjects is a relevant topic in societies characterized by the progressive aging of their populations [7]. With telemedicine, neuropsychologists can use, instead of the classical face-to-face (FTF) interview, computers, digital tablets, and/or a handheld or another digital interface to administer, score, and interpret the tests measuring cognitive abilities and related factors [8].

If we look at computer-based cognitive tools, they are commonly used as productivity software. Applications including semantic networks, spreadsheets, expert systems, and databases function as computer-based cognitive tools [9]. Subjects affected by dementia may benefit from diagnosis, as well as therapy monitoring, that overcomes the difficulties of their transfer, particularly if they live in remote rural areas, in difficult-to-access areas, and/or are far from health institutions [10]. The AD assessment via telemedicine can also increase the capacity to gather a large number of individuals in a short time, reduce waiting lists and assessment times, and increase the accessibility for patients living in remote areas.

Previous studies have shown no main differences in the results of the Mini-Mental State Evaluation (MMSE) test, the most common tool for preliminary cognitive screening, if administered via telehealth or FTF [11,12]. Other neuropsychological tests investigated in telemedicine were ADAS-Cog [13], clock drawing [14], Oral Trails [15], the Boston naming test, the picture description test (auditory response version), the Boston Diagnostic Aphasia Examination (BDAE) [16], letter and category fluency [17], Digit Span [18], Repeatable Battery for the Assessment of Neuropsychological Status [18,19], the Hopkins Verbal Learning Test-Revised (HVLT-R), IQCODE [20], the Aural Comprehension of Words and Phrases (ACWP) and Controlled Oral Word Association Test [21], and the Rowland Universal Dementia Assessment Scale (RUDAS) [22].

The possibility of receiving care and of being monitored remotely can be useful for assisting older people in remote or difficult-to-access areas or with difficulties to move from home due to pathologies or lack of caregivers. Even in urban regions, AD patients and caregivers may have difficulties leaving home because there is not enough availability of appropriate transportation. On the other hand, dementia experts cannot always be available near home. In this respect, telehealth may make access to dementia care and education easier without the need to leave home, and may significantly reduce exits for medical examinations before it turns out to be hard to handle [23,24].

It is accepted that neuropsychological investigations of the cognitive status of old people can be done by telemedicine, but doubts exist on the reliability of the tests applied to assess cognitive status via information and communication technologies (ICT). The purpose of this review was to investigate, through a narrative synthesis of the scientific literature, the reliability of telemedicine approaches in the diagnosis and treatment of adult-onset dementia disorders and to identify possible grey areas needing to be further defined. Moreover, this work has reported the comparison of neuropsychological assessment by videoconference with FTF evaluation.

## 2. Methods

### 2.1. Document Search

The literature search was performed via two major medical databases: PubMed (MEDLINE) and the Cumulative Index to Nursing and Allied Health Literature (CINAHL). The review was carried out in March 2021 and it was carried out according to the Preferred Reporting Items for Systematic Reviews and Meta-Analyses (PRISMA) guidelines. PRISMA is an evidence-based minimum set of items for reporting in systematic reviews and meta-analyses [25]. The search keywords included ‘telemedicine’, ‘Alzheimer’s disease or AD or dementia’, ‘Mini-Mental State Examination (MMSE)’, ‘cognitive evaluation’, ‘neuropsychological assessment’, ‘Stroop test’, and ‘teleconference’. Moreover, the Boolean AND operator were used and reported the following search strings: ‘telemedicine in Alzheimer’s disease’, AND ‘MMSE by telemedicine’, AND ‘dementia patient analysis in COVID-19 via telemedicine’, AND ‘cognitive evaluation by telemedicine in Alzheimer’s disease’, and ‘neuropsychological assessment’.

### 2.2. Inclusion and Exclusion Criteria

This review included research articles published in the last 20 years (2001–2020). The inclusion criteria for document selection were the English language, at least one keyword corresponding to the above entries in the title or abstract, clinical trials comparing telemedicine with other traditional tools, and participants affected by dementia and/or cognitive impairments. Only papers where the cognitive evaluation was the primary end-point were selected. Groups of particular study types such as analytical studies, meta-analysis, clinical trials, and randomized controlled trials (RCT) were included as well. The studies with different characteristics other than the inclusion criteria and items that did not deal with telemedicine applications for neurodegenerative diseases were excluded. Articles were excluded from further evaluation if they met any of the following criteria: (1) not in the English language, (2) with limited text availability, (3) works published before 2001, and (4) review articles.

### 2.3. Quality Assessment

The Newcastle-Ottawa Scale (NOS) was used to carry out a quality evaluation of filtered papers. NOS scores are used to assess the quality of non-randomized studies in meta-analyses, based on factors like structure, convenience, overall quality, and suitability for review studies. Quality assessment was evaluated based on final NOS scores of each study: poor (if score 0–3), modest (4–6), and excellent (7–9). Studies that passed the quality test with NOS ≥ 7 were considered suitable for this review.

## 3. Results

### 3.1. Search Outcomes

The document search resulted in 1253 documents, including 934 items from PubMed and 319 items from CINAHL. Because of duplication, 764 items were excluded during the initial screening. The remaining 478 publications were further investigated to assess their adherence to the inclusion and exclusion criteria. From this first screening, 68 papers were eligible. These publications were distributed to all authors to perform quality assessment tests.

**Figure 1:**
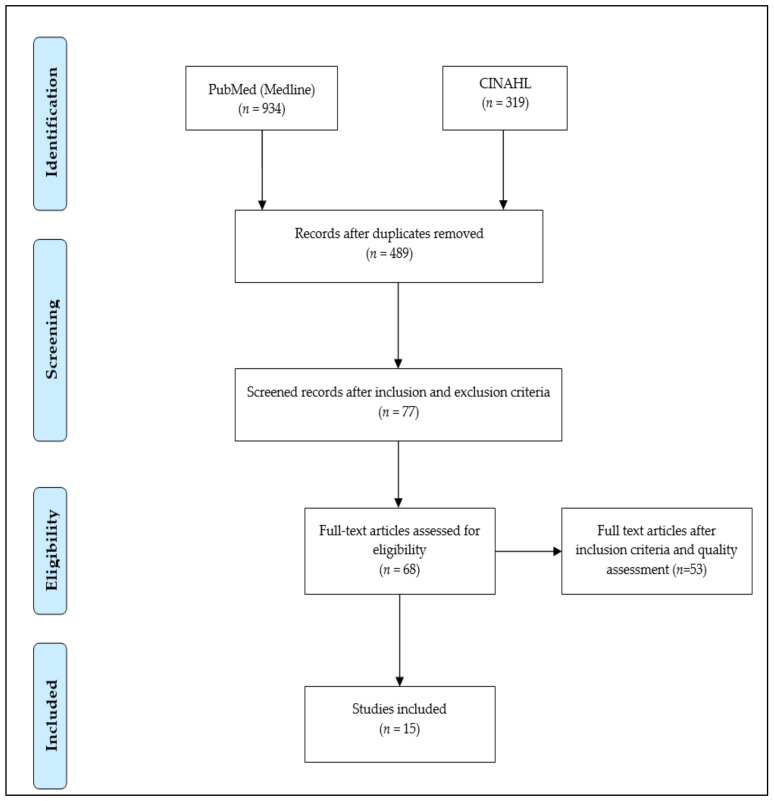
Study selection explained via PRISMA [26] flow chart.

Figure 1 summarizes the steps involved in document filtering. The authors carried out a first independent evaluation by reading the abstracts and consequently drafting a list of the articles considered eligible. From the evaluation analysis performed by single co-authors, possible divergences in the evaluation were considered. When one divergence was individuated, the different points of view were discussed to reach an agreement. Once the authors decided which articles should be included, the full articles were read to gather data useful for the research. The information evaluation was carried out independently and opinions were compared to formulate a final consensus. At the end of this preliminary analysis, 15 studies satisfying the inclusion criteria were further considered.

### 3.2. Study Findings 

The selected papers were further separated into two groups based on their characteristics. Eight studies [13,19,27,28,29,30,31,32] selected according to the criteria indicated above compared several neuropsychological tests administered in FTF versus videoconference modality. Studies highlighting the AD screening via telemedicine are reported in Table 1 and telemedicine studies with other neurological tests are presented in Table 2.

#### 3.2.1. MMSE-Based AD Screening

The Mini-Mental State Examination (MMSE) test is one of the AD screening tools in cognitive neurology. In this review, six studies were associated with AD identification with the MMSE rating (between 0 and 30) and compared outcomes with dual mode of communication (FTF and videoconference). For instance, in Reference [13], the authors investigated 28 AD patients, including 8 males (age: 73.88 ± 7.45) and 20 females (age: 76.00 ± 5.40) recruited and followed in the Alzheimer’s Unit of the A Cardarelli National Hospital (Naples, Italy) at baseline and after 6, 12, 18, and 24 months of observation. Patients were first evaluated FTF by a psychologist and then, after 2 weeks, by another psychologist via videoconference. It was found that comparison is feasible when subjects have non-serious cognitive impairment (MMSE > 15).

Another study investigated 69 subjects (age: 74.90 ± 9.46) for the assessment of cognitive performance using the Telephone Interview for Cognitive Status-Modified—Portuguese version (TICSM-PT) by video teleconference (VTC), telephone, and in-person approaches. Correlation analyses showed a high association between the testing modalities of TICSM-PT VC and TICSM-PT telephone (r = 0.885), and TICSM-PT VC and MMSE FTF (r = 0.801). Using the previously validated threshold for cognitive impairment on the TICSM-PT telephone, TICSM-PT VC administration presented a sensitivity of 87.8% and a specificity of 84.6% [33]. The comparative study on AD patients from both rural and urban settings in the objective of the MMSE evaluation by videoconference and FTF reported similar MMSE scores via videoconference (mean: 27.6 ± 3.10) and face-to-face (mean: 27.6 ± 3.09) modalities. The results obtained were consistent in subjects with or without cognitive impairment and people with MMSE scores as low as 15 [27]. Another study on 202 patients, 41% of which were affected by mild cognitive impairment (MCI), compared the traditional and videoconference modality to evaluate memory, executive functions, constructive abilities, orientation, verbal fluency, and attention. It was reported that the reliability of in-person administrated MMSE presented a slight difference between MMSE by videoconference (22.70 ± 6.51) and FTF (22.34 ± 6.35). The precision of the lower (−6.41 to −4.36) and the upper (3.62 to 5.67) limits of agreement for the 95% confidence interval was narrow, demonstrating a sufficiently large sample size [35].

The comparison between standard MMSE and videoconference-based MMSE (V-MMSE) by conducting experiments on 342 subjects was done in [34]. The authors reported high sensitivity and specificity for V-MMSE and an accuracy of 0.96 (95% CI: 0.94–0.98). Intra/RR and inter/RR were highly significant. Another investigation to develop telemedicine protocols including the V-MMSE and FTF MMSE for AD diagnosis presented the conventional MMSE (mean: 23.3 ± 3.6) and V-MMSE (mean: 24.2 ± 3.7). No significant differences emerged between the standard MMSE and the videoconference MMSE [28]. Another experimental study on 20 AD patients (mean age of 82 years) evaluated cognitive functions by MMSE through remote and FTF administration. The remote evaluation provided results similar to direct assessments in 60% of patients. The average MMSE score by remote assessment was 24.0 (range 11.0–30.0) and 24.3 (range 9.0–30.0) by direct assessment. The correlation between direct and remote MMSE scores was 0.90. However, there was a moderate difference between FTF and remote assessments in 8 of 20 participants (40%) of two points or more on the MMSE [36].

#### 3.2.2. Other Neuropsychological Tests

Neuropsychological tests were applied to a large extent to assess psychological function, which is linked to specific brain structure [40]. These tests are used for brain research and medical settings for the diagnosis of deficits. Some studies are emphasized to conduct them via telemedicine or teleconference. Over the past 18 months, the world has been experiencing a severe pandemic caused by the novel coronavirus disease (COVID-19) and elder patients with mild dementia have been confined to home. Such scenarios raise the importance of telemedicine with television-based integrated approaches. In Reference [41], the authors suggested that during COVID-19 confinement, the mental, behavioral, and physical health of older adults is at risk.

For instance, an investigation on the diagnosis of MCI was performed through a brief battery of standard neuropsychological tests (mentioned in previous sections), administered via video teleconference (VTC) compared with traditional FTF administration [29]. The authors reported no significant differences between the clock drawing test, Digit Span Backwards, Oral Trails B, HVLT-R scores, or verbal and category fluency. However, small statistically significant differences were seen on Digit Span Forward, Oral Trails A, and BNT total, but they were not clinically meaningful.

The feasibility and reliability of the Repeatable Battery for the Assessment of Neuropsychological Status (RBANS) administration, comparing videoconference with FTF conditions with and without cognitive impairment, was tested [19]. The correlation of the total score obtained by FTF and video teleconference was significantly high. Similar mean scores of the RBANS index between the two assessment conditions were also found. Another work on evaluating the validity of the dementia diagnosis via videoconference and outlined videoconference found it was no lesser than that of an FTF assessment [30].

On other hand, vascular cognitive impairment (VCI) indicates disfunction of cognitive abilities with a hidden vascular etiology. It is entirely expected in the elder population and is especially prevalent in patients with stroke. For that, the Montreal Cognitive Assessment (MoCA) is considered as an instrument for clinical screening of VCI. In Reference [38], the authors conducted a 5 min protocol of MoCA administration over the telephone. They concluded MoCA protocol is free and investigated its reliability, validity, and feasibility through a four-item examination including verbal learning and memory, examining attention, orientation, and executive functions/language. Besides, telephone interviews for cognitive status (TICS) and Telephone-MoCA (T-MoCA) are performed well in MCI detection after stroke and discriminability are more prominent when there is proof of hindrances in different cognitive domains versus a single domain [39].

In Reference [31], the authors investigated five language tasks on 10 patients with mild AD by telemedicine and FTF analysis. On each of the five language tasks, the Wilcoxon signed-ranks test indicated no significant difference in performance between the telemedicine and in-person conditions for each participant. Similarly, the comparison of VC-based diagnostic interviewing with the conventional FTF diagnosis was done in [32]. The authors administered a brief battery of common neuropsychological tests through tele-cognitive and traditional FTF approaches, and the neuropsychology assessment included the MMSE, Hopkins Verbal Learning Test-Revised (HVLT-R), Digit Span, category fluency, letter fluency (FAS and CGL version), and a 15-item version of the Boston naming test (BNT). Measures of MMSE (*p* value = 0.89), Digit Span (*p* value = 0.81), category fluency (*p* value = 0.58), letter fluency (*p* value = 0.83), and BNT (*p* value = 0.88) showed excellent agreement between videoconference and FTF testing.

## 4. Discussion

This work was focused on the reliability of telehealth technologies in AD assessment. Telemedicine or teleconferencing services are expanding the limits of the provider act by reducing or eliminating travel barriers and by making admittance to medical care simpler. The incorporation of telehealth technologies in AD assessment can promote various treatment approaches. This systematic review summarized tele-neuropsychological studies highlighting the AD screening and comparison of both approaches via telemedicine and FTV. As far as we know, there are only a few reviews in the literature dealing with the use of telemedicine in the diagnosis of dementia [14,15,16,17,34,35,36].

MMSE via videoconference is as accurate as FTF examination and can be used alternatively to it. However, a study has reported that comparison is not feasible when subjects have serious cognitive impairment (MMSE < 15) [35]. In the European countries where the V-MMSE has been validated to diagnose AD, a high concordance was found between the diagnosis achieved traditionally and by videoconference [42]. Internet broadband with an internet video camera and a personal computer are the only facilities required. Studies with the AD screening with V-MMSE did not find differences between scores obtained when the MMSE was administered FTF or in a videoconference. Subjects were old adults with dementia or MCI. Only a single study [34] found that the maximum score at the MMSE should be 28 instead of 30 if the test was administered via videoconference. Another work found that the MMSE ranking by videoconference is two points lower than MMSE FTF in 40% of subjects [36].

The study carried out by Carotenuto et al. [13] was probably the first study that investigated the ADAS-Cog test remotely. This test is widely used in clinical trials on pharmacological agents used or investigated for their effectiveness in the symptomatologic treatment of dementia disorders. This was likely due to the patients’ difficulty in understanding the meaning of some test questions. The authors suggested that videoconferencing techniques can be effectively used in patients with mild to moderate dementia, whereas they should be excluded in patients with moderate to severe dementia. The feasibility and reliability of this test by telemedicine should be confirmed by further studies.

Studies with other neuropsychological tests administered by telemedicine have examined the two modalities of administration (FTF and videoconference) of some memory tests, executive test language, and others. It was found that the scores obtained through videoconference corresponded to those of the FTF assessment. Small differences were found in the scores of some tests including the clock drawing test, digits span forward (but not digits backward), the Hopkins Verbal Learning Test-Revised (HVLT-R) [27], Oral Trail part A, and the Boston naming test (BNT) [29].

Despite the advantages in tele-neurology, further improvements are challenged by the financial environment. Technical improvements are largely facilitated the tele-neurology practice, but maintaining connectivity is a challenge alongside the need for valid informational technical resources that also add a substantial component to the cost of tele-neurology [43]. If audio or video is disabled during patient monitoring, a back-up plan is needed for an adequate assessment. Besides, getting licensing and authentication of telemedicine can be time consuming and often laborious, which adds extra administrative cost and burden. Finally, some scoring procedures in the videoconference modality should be standardized to ensure their reliability [44]. It is important enough training in the technical, legal, and clinical intricacies of videoconference assessment before engaging in remote assessment [45].

Patient acceptance and satisfaction of the telemedical assessment are mostly reported, with subjects being confident in it, and also in the protection of their privacy. Increased convenience for neuropsychological analysis via video/teleconference is also appreciated more with time [46]. In summary, standard neuropsychological tests can be used by videoconference provided that some modifications in the instructions and in evaluating scores are considered. To ensure reliable results in this, field tests must be administered by raters who are properly trained and with specified competencies [32,47,48]. One of the possible limitations of this work is that we analyzed only two medical databases (PubMed or MEDLINE and CINAHL) using specific strings. Consequently, not all the eligible papers may have been identified. Moreover, the variability in neuropsychological measures variability in statistical assessments can limit the findings of the present study.

## 5. Conclusions

In conclusion, this review provides support for telemedicine’s reliable use in the screening of adult-onset dementia disorders including MCI and in the evaluation of AD in mild to moderate stage subjects and in subjects to be diagnosed. It can also represent an easy modality of assessment of patients undergoing pharmacological or other types of treatment. Telemedicine, by reducing the global costs of the assessment and the cognitive follow up of older adults, could represent an opportunity for making evaluation and care of adult-onset cognitive dysfunctions more affordable for public health systems and families of patients suffering from these problems. Because of the feasibility and of the relatively easy use of these technologies, they can be useful for a personalized medicine approach for the treatment of adult-onset dementia disorders.

## Figures and Tables

**Table 1:** Articles comparing MMSE test for Alzheimer’s disease by telemedicine versus face-to-face examination.

N	Study Type	Participants	Telemedicine Mode	Delay between Testing Modalities	Findings	Ref
1	Pilot	28	VTC	6, 12, 18, and 24 months. The interval between each administration was 2 weeks	No differences in the MMSE and ADAS-cog scores when the tests were administered FTF or by videoconference MMSE of mean ± SD reported for face-to-face examination (13.9 ± 4.9), ADAS-cog (9.0 ± 3.8), videoconference (42.8 ± 12.5), and ADAS-cog mean (56.9 ± SD 5.5).	[13]
2	Pilot	69	Telephone and VTC	1 month after the MMSE FTF assessment; 2-month interval from the VTC administration	A strong association between the TICSM-(Portuguese version) applied by videoconference and by telephone (r = 0.885), and between them and the MMSE FTF (r = 0.801)	[33]
3	Clinical trail	202	VTC	Same day	MMMSE administered via VTC and FTF was comparable (with the score is >15). The correlation of score obtained by FTF and video teleconference of Repeatable Battery for the Assessment of Neuropsychological Status (RBANS) administration was significantly high (mean: 0.80).	[27]
4	Experimental	342	VTC	Within 6 weeks	VMMSE is comparable with MMSE FTF, but with the cut-off at 28.	[34]
5	Clinical trail	71	VTC	6 and 7 weeks	No difference between VMMSE and face-to-face (*p* = 0.223) examinations.	[35]
6	Longitudinal	20	VTC	-	The agreement between FTF and videoconference indicates that telemedical assessment is valid to diagnosed AD. The mean MMSE FTF was 23.3 (SD 3.6), VMMSE by videoconference was 24.2 (SD 3.7).	[28]
7	Experimental	20	VTC	-	MMSE by videoconference and FTF yielded similar results in 60% of patients. However, there was a moderate difference in 40% of two points or more on the MMSE.	[36]

**Table 2:** Neuropsychological tests other than MMSE for AD by telemedicine versus face-to-face examinations.

N	Study Type	Participants	Telemedicine Mode	The Delay between Testing Modalities	Findings	Ref
1	Survey	108	Television and telephone	March 25 to 6 April 2020	No significant differences were found in health and well-being among the control and intervention and groups. Participants with TV-Assist Dem performed more memory exercises (24/93, 52% vs. 8/93, 17.4%; *p* < 0.001) than control respondents.	[37]
2	Pilot	84	VTC	Same day	Good feasibility and reliability of videoconference administration for the clock drawing test, Digit Span Forward and Backward, Oral Trails, Hopkins Verbal Learning Test-Revised, letter and category fluency, and a short-form Boston naming test.	[29]
3	Pilot	18	VTC	Same day	The correlation of score obtained by FTF and video teleconference of Repeatable Battery for the Assessment of Neuropsychological Status (RBANS) administration was significantly high (mean: 0.80).	[19]
4	Pilot	205	VTC	-	The difference in agreement between the clinical practice group (Po: 70%) and the video group (Po 71%) was 1% (0.01 CI 0.12–0.13)	[30]
5	Experimental	1013	Telephone	30 days	Total scores of the MoCA and MoCA 5-min protocol were highly correlated (r = 0.87, *p* < 0.001) and MoCA 5 min protocol positively correlated with education (0.41, *p* < 0.001) and negatively with age (r = −0.36, *p* < 0.001)	[38]
6	Pilot	108	Telephone	1 week	For TICS, the area under receiver operating characteristic (AU-ROC) curve values ranges from 0.76 to 0.83 and for T-MoCA the AU-ROC values range from 0.73 to 0.94. It is approved as both TICS and T-MoCA are valid screening tools for multidomain MCI through multiple test definitions.	[39]
7	Experimental	10	VTC	Same day	On the picture description test, BNT, Token test, and the Aural Comprehension of Words and Phrases (ACWP) and Controlled Oral Word Association Test, the Wilcoxon signed-ranks test indicated no significant difference in performance between the videoconference and face-to-face evaluations.	[31]
8	Clinical trail	33	VTC	Same day	Measures of digit span (*p* = 0.81), category fluency (*p* = 0.58), letter fluency (*p* = 0.83), and BNT (*p* = 0.88) showed excellent agreement between tele-cognitive and face-to-face testing. Global validity of tele-cognitive assessment.	[32]

## Data Availability

Not applicable.
